# Editorial: Asthma: physiology and pathophysiology

**DOI:** 10.3389/fphys.2024.1403211

**Published:** 2024-04-08

**Authors:** Carmen Silvia Valente Barbas

**Affiliations:** Pneumology, INCOR, University of São Paulo, São Paulo, Brazil

**Keywords:** asthma, pathophysiology, pulmonary function, obesity, treatment

Asthma, the most prevalent lung disease globally, is complex and multifactorial. Advances in understanding of its pathophysiology allow a better classification of different subtypes that permit a multidisciplinary approach for asthma diagnosis and treatment (Venkatesan, 2023)^.^

Asthma patients have a history of variable respiratory symptoms: wheeze, shortness of breath, chest tightness and cough, and confirmed variable expiratory airflow limitation. The patient must have airflow obstruction and bronchial inflammation and abnormal mucus production and clearance (Venkatesan, 2023). Mucus plugs develop in patients with severe asthma, and the magnitude of the mucus plug score correlates with the degree of airway obstruction. Mucus plugs are associated with distal deficits in regional ventilation, which can be delineated by hyperpolarized gas magnetic resonance imaging. Lung imaging technologies such as thoracic tomography during inspiration and expiration can add diagnostic insight pertaining to bronchial, air-trapping and hyperinflation, and possible bronchiectasis (Krings and Gierada, 2023).

Asthma is characterized by Type II inflammation, including Th2 lymphocytes that produce interleukins 3,4,5, and 13 and GM-CSF. Th2 lymphocytes also express the chemokine receptors CCR4 and CCR8 and the chemoattractant receptor CRTH2m, a receptor for prostaglandin D2, suggesting interactions between mast cells and chemotactic signals that target eosinophils and Th2 cells that possibly sustain bronchial inflammation (Venkatesan, 2023). In this Research Topic, Drake et al. report that asthmatic lung fibroblasts promote type 2 immune responses via an endoplasmic reticulum stress response dependent on thymic stromal lymphopoietin secretion.

Obesity can increase the frequency and severity of asthma exacerbations, especially in female patients (Tashiro et al., 2024). In this Research Topic, Wang et al. report that body mass index affects spirometry indices in patients with asthma.

In their paper, Zhang et al. report an association between abdominal obesity with lung function, FeNO, and blood eosinophils in adults with asthma. The authors show that general and abdominal obesity were associated with lung function impairment and a significant reduction of FeNO and blood eosinophil percentage, suggesting the importance of concurrent determination of body mass index and waist circumference in asthma clinical practice.

Differential diagnosis of asthma varies with age and can vary from an inhaled foreign body in a young child to pulmonary embolism or cardiac failure in older people. Comorbidities must also be assessed, as arterial hypertension, diabetes, tobacco smoking, gastro-esophageal reflux, chronic rhinosinusitis, psychological - psychiatric alterations, and coronary artery disease can all interfere with the symptoms and treatment of asthmatic patients (Venkatesan, 2023).

According to the Global initiative for asthma (GINA- update 2023), the best treatment for adults with asthma involves a five-step algorithm that is initiated in step 1 with a low dose ICS-formoterol as needed and finishes with step 5 in which the patient can use high ICS- formoterol plus a LAMA and short course of oral corticosteroids (Venkatesan, 2023). In this Research Topic, Umeda et al. report real-world effects of once-daily inhaled steroids (fluticasone furoate)combined with long-acting beta-2 agonist (vilanterol) and long-acting muscarinic antagonist (umeclidinium) on lung function tests of asthma patients in Japan. The authors conclude that once-daily fluticasone/vilanterol/umeclidinium (200/62.5/25 μg) was effective against asthma without serious adverse events. In Step 5 the patient can also be referred for phenotype assessment for possible biological therapy. Recently, Pavord et al. presented robust data supporting fractional exhaled nitric oxide (FeNO) as a clinically viable prognostic biomarker for accelerated lung function deterioration (LFD) and predicted the treatment response to dupilumab in asthma. Additional research is needed to establish patterns of LFD in patients with moderate-to-severe asthma, as well as the prognostic and predictive role of FeNO (Pavord et al., 2024). Recently, Tezepelumab was associated with a reduction in occlusive mucus plugs versus placebo in a randomized controlled trial in patients with moderate-to-severe uncontrolled asthma. In Tezepelumab recipients, reductions in mucus plug scores were correlated with improvements in lung function and reductions in blood eosinophil count and levels of eosinophil-derived neurotoxin, a biomarker of eosinophilic degranulation (Nordenmark et al., 2023).

## References

[B1] Krings J. G. Gierada D. S. (2023). Do biologic therapies decrease mucus plugging in asthma? NEJM Evid. 2 (10), EVIDe2300179. Epub 2023 Sep 26. PMID: 3832018. 10.1056/EVIDe2300179 38320185 PMC12288139

[B2] Nordenmark L. H. Hellqvist Å. Emson C. Diver S. Porsbjerg C. Griffiths J. M. (2023). Tezepelumab and mucus plugs in patients with moderate-to-severe asthma. NEJM Evid. 2 (10), EVIDoa2300135. Epub 2023 Sep 20. PMID: 38320181. 10.1056/EVIDoa2300135 38320181

[B3] PavordI. D.de Prado GómezL.BrusselleG.JacksonD. J.BrightlingC. E.PapiA. (2024). Biomarkers associated with lung function decline and dupilumab response in patients with asthma. Am. J. Respir. Crit. Care Med. 8. Online ahead of print. PMID: 38329781. 10.1164/rccm.202310-1751LE 38329781

[B4] Tashiro H. Kurihara Y. Kuwahara Y. Takahashi K. (2024). Impact of obesity in asthma: possible future therapies. Allergol. Int. 73 (1), 48–57. 10.1016/j.alit.2023.08.007 37659887

[B5] Venkatesan P. (2023). 2023 GINA report for asthma. Lancet Respir. Med. 11 (7), 589. PMID: 37302397. 10.1016/S2213-2600(23)00230-8 37302397

